# Application on Gold Nanoparticles-Dotted 4-Nitrophenylazo Graphene in a Label-Free Impedimetric Deoxynivalenol Immunosensor

**DOI:** 10.3390/s150203854

**Published:** 2015-02-06

**Authors:** Christopher Edozie Sunday, Milua Masikini, Lindsay Wilson, Candice Rassie, Tesfaye Waryo, Pricilla G. L. Baker, Emmanuel I. Iwuoha

**Affiliations:** Sensor Laboratory, Chemistry Department, University of the Western Cape, Private Bag X 17, Bellville 7535, South Africa; E-Mails: csunday@uwc.ac.za (C.E.S.); masikinimilua@gmail.com (M.M.); 2724554@myuwc.ac.za (L.W.); 2734778@myuwc.ac.za (C.R.); twaryo@uwc.ac.za (T.W.); pbaker@uwc.ac.za (P.G.L.B)

**Keywords:** deoxynivalenol, mycotoxin, graphene, immunosensor, graphene oxide, Nafion

## Abstract

In this paper, we report a new concept to construct a label-free electrochemical inhibition-based immunosensor for the detection of the mycotoxin deoxynivalenol (DON) in cereal samples. The electrochemical impedance spectroscopy of tris(bipyridine) ruthenium (II) chloride was used as a marker enhanced with gold nanoparticles-dotted 4-nitrophenylazo functionalized graphene (AuNp/G/PhNO_2_) nanocatalyst mediated in Nafion on a glassy carbon electrode. Under the optimized conditions, the formation of immunocomplexes inhibited electron flow and increased the charge transfer resistance of the sensing interface linearly. The change in impedance was proportional to DON concentrations in the range of 6–30 ng/mL with a sensitivity and detection limit of 32.14 ΩL/ng and 0.3 μg/mL, respectively, which compares favorably with the ELISA result. The proposed sensor had a stability of 80.3%, good precision and selectivity in DON standard solution containing different interfering agents, indicating promising application prospect for this strategy in designing impedimetric, electrochemiluminescent, voltammetric or amperometric sensors.

## 1. Introduction

**Figure 1.**
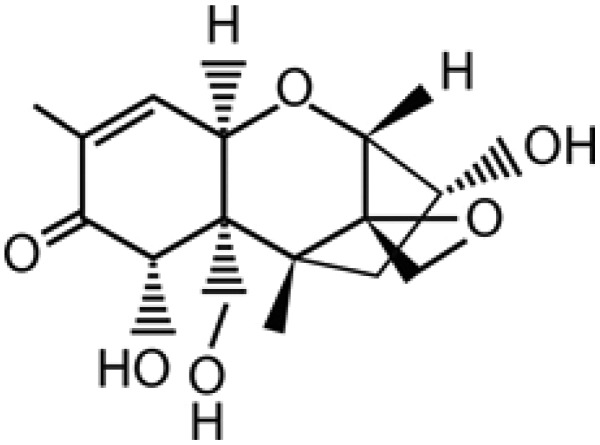
Chemical structure of the mycotoxin deoxynivalenol.

The mycotoxin deoxynivalenol (DON) is a low molecular weight metabolite and chemical by-product of the fungal species *Fusarium graminearum* and *Fusarium culmorum* which occur naturally in the soil of crop fields and contaminate a wide range of crop plants before and after harvest [1]. It belongs to a class of mycotoxins called the trichothecenes which are commonly found in cereals or cereal-based food and feedstuffs. They are classified into group A and group B compounds, depending on their structure. The most important types of group A-trichothecene are T-2 toxin and HT-2 toxin. DON falls within the group B-trichothecenes [2]. DON often co-exists with other mycotoxins such as zearalenone and nivalenol. Its levels range from μg/kg to tens of mg/kg and vary year-to-year depending on the climate, season, geographic conditions and agronomic practice. The deoxynivalenol structure (Figure 1, [1]) is characterized as a tetracyclic sesquiterpene with seven stereocentres, and six oxygen atoms which would allow for multiple hydrogen bonding, including an epoxide, a carbonyl, cyclic ether and three alcoholic OH groups [3,4].

Deoxynivalenol is toxic, but it has not been reported as having carcinogenic, teratogenic or mutagenic properties [5–7]. The most important structural features responsible for the biological activities of DON are the 12,13-epoxy ring, the presence of hydroxyl/acetyl groups and their position in the chemical structure of DON. Other mycotoxins like T-2 toxin, HT-2 toxin and nivalenol (NIV) also have the same effect, but it appears that they differ in their toxic capacities and it is not clear whether they work via identical mechanisms at a cellular level [4]. The acute effects of DON in animals and humans include reduced feed uptake, nausea, vomiting, diarrhoea, abdominal pain, headache, dizziness, fever, skin irritation and immunosuppression. No human deaths have been attributed to DON yet. Humans are directly exposed to these risks through foods of plant origin (cereal grains) or indirectly through foods of animal origin (kidney, liver, milk, eggs) [5–7].

DON is an important toxin of cereal foods and constitutes an increasing problem in several countries because the occurrence of the fungal species that produce it is very high in Nature. The growth of these fungi is season dependant and complete prevention is impossible. Because of concerns with the increasing toxic effects of DON on livestock and humans, the United States Department of Agriculture (USDA), Food & Drug Administration (FDA) and European Community have instituted advisory levels of 1 ppm for wheat products for human consumption, 5 ppm of grain products for most animal feeds and 10 ppm of grain products for cattle feed [5,6]. The European Union (EU) has also established ranges from 200 to 1750 μg/kg depending on the kind of cereal and cereal products. A provisional maximum tolerated daily intake (PMTDI) of DON for 1 μg/kg body weight (BW) was established by the World Health Organization Joint Expert Committee on Food Additives (WHOJECFA) on the basis of NOAEL (NOAEL = no observed adverse effect level) [8,9].

The analytical methods reported for the determination of DON include PCR, GC, GC-MS, HPLC, thin-layer chromatography and enzyme-linked immunosorbent assay (ELISA) [10–16]. Some of these methods allow good accuracy of quantification and good detection limits, but they are not cost effective; some suffer from low selectivity and usually require significant amounts of time associated with labour-intensive sample cleanup, sophisticated instrumentation, skilled operators or technical expertise. In view of these analytical challenges, a gold nanoparticles-dotted 4-nitrophenylazo functionalised graphene (AuNp/G/PhNO_2_) composite was used to develop a sensor platform by applying Nafion 117 as a binder and incorporating [Ru(bpy)_3_]^2+^ as a cationic reactant on a glassy carbon electrode (GCE) [17]. The diazonium-modified platform was adopted in the construction of easy to use, rapid, cost effective and signal enhanced immunosensor for the detection and determination of DON levels in cereal food items via an impedimetric system. The formation of immunocomplexes between DON antibody and DON antigenic species to which it selectively binds inhibited the electron flow and increased the charge transfer resistance of the sensing interface linearly, with the change in impedance being proportional to DON concentration.

## 2. Experimental Section

### 2.1. Chemicals and Reagents

Deoxynivalenol antigen, DONag (formula weight, 296.32 g/mol) was purchased from Sigma-Aldrich (St. Louis, MO, USA; product code 01567-5MG), IgG1 monoclonal antibody of deoxynivalenol (DONab, 1 mg) was purchased from Antibodies-online (Aachen, Germany, order number ABIN1022125), ELISA kit (Veratox 5/5 for deoxynivalenol: 0.1 ppm) was purchased from Neogen Corporation (Lansing, MI, USA), certified corn, wheat and roasted coffee reference materials were purchased from RIDASCREEN^®^ (Washington, MO, USA). Methanol (CH_4_O, HPLC grade), tris-ruthenium 2,2-bipyridine chloride (Ru(bpy)_3_Cl_2_), ethanol (C_2_H_6_O, analytical grade), bovine serum albumin (BSA), 1-ethyl-3-(3-dimethylaminopropyl) carbodiimide hydrochloride (EDC), N-hydroxy-succinimide (NHS), basic salts including sodium phosphate dibasic (Na_2_HPO_4_), sodium phosphate monobasic (NaH_2_PO_4_), potassium Chloride (KCl) used in the preparation of 0.1 M phosphate-buffered saline (PBS pH 7.2), were all purchased from Sigma-Aldrich (Johannesburg, South Africa). Nafion 117 solution purchased from Fluka (Johannesburg, South Africa). Gold nanoparticles-dotted 4-nitrophenylazo functionalised graphene (AuNp/G/PhNO_2_) and 4-nitrophenylazofunctionalised graphene (G/PhNO_2_) were synthesised as described elsewhere [17]. All other chemicals were of analytical grade, Milli-Q de-ionized water (resistance over 18.2 MΩ cm) purified by a Milli-QTM system was used for aqueous solution preparations throughout. Analytical grade argon from Afrox (Cape Town, South Africa) was used to degas the system.

### 2.2. Apparatus

All cyclic voltammetry and chronoamperometry experiments were recorded with BASi 100B electrochemical work station (LG Fayette, TX, USA). The electrochemical impedance spectroscopy measurements were carried out using Voltalab PGL 402 from Radiometer Analytical (Lyon, France) and the data plotted in the form of complex plane diagrams (Nyquist plots). The conventional three-electrode system was adopted where the working electrode used was glassy carbon electrode (surface area = 0.071 cm^2^), the counter electrode was platinum wire (diameter 1.0 mm) and all potentials mentioned in all the experimental results were referred to standard silver/silver chloride (saturated KCl solution) electrode (Bioanalytical Systems Ltd., Warwickshire, UK). The electrolytes used were measured into 20 mL electrochemical cell and de-aerated with argon gas for at least 10 min before measurements. An argon atmosphere headspace was maintained throughout the duration of all the experiments.

### 2.3. Pre-Cleaning of Working Electrode

Prior to electrochemical modifications, glassy carbon electrode (GCE) was polished consecutively with aqueous slurries of 1.0, 0.3 and 0.05 micro alumina powder, for 1 min on a micro-cloth pad (Buehler, Düsseldorf, Germany) and rinsed thoroughly with doubly distilled water between each polishing step. Residual polishing material was removed by washing successively with 1:1 water to nitric acid solution, ethanol and doubly distilled water in an ultrasonic bath, air dried and used immediately.

### 2.4. Preparation of Standard Solutions

DONag was dissolved in 1 mL of analytical grade methanol to give 5 mg/mL (0.0169 M) stock solution and stored as 250 μL aliquots each in tightly sealed vials at −20 °C. Before every experiment, 1 × 10^−3^ M and 1 × 10^−6^ M of DONag standard solutions were prepared in a (3:1) mixture of methanol/PBS solution, hereafter referred to as methanolic phosphate buffer saline (MPBS) solution, and used for further dilutions. The DONab (1 mg) was reconstituted by adding 1 mL deionised water to give 1 mg/mL stock solution and stored as 250 μL aliquots each in tightly sealed vials at −20 °C.

### 2.5. Sample Preparation and Extraction

Following the extraction procedure described by Veratox Elisa Kit manual, 10 g of ground certified corn, wheat and roasted coffee reference materials were each weighed out into separate 250 mL capped pre-cleaned flask, mixed with 100 mL of deionised water and shaken vigorously using hand for 3 min. The mixture was allowed to settle for 2 min and then filtered using a Whatman #1 filter paper. The filtrate was collected for analysis without further preparation.

### 2.6. Preparation of G/PhNO_2_ and AuNp/G/PhNO_2_

**Figure 2.**
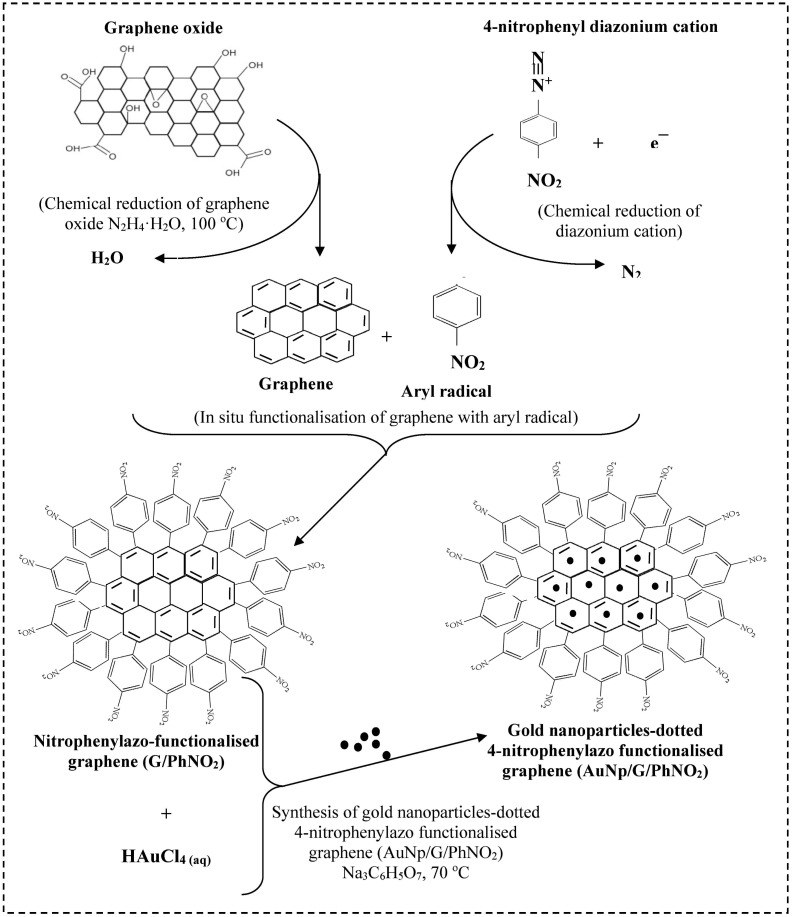
The illustration for simultaneous chemical reduction of diazonium cation to diazonium radical, GO to graphene, in situ PhNO_2_ functionalisation of graphene and the immobilization of gold nanoparticles onto nitrophenylazo functionalized graphene surface.

G/PhNO_2_ and AuNp/G/PhNO_2_ were chemically synthesized as described previously [17]. Briefly, Graphene oxide (GO) was reduced in three stages: pre-reduction of GO, functionalisation of pr-reduced GO and post-reduction to give G/PhNO_2_. This was functionalized further with gold nanoparticles to give AuNp/G/PhNO_2_ (Figure 2 [17]).

### 2.7. Preparation of Sensor Platform

**Figure 3.**
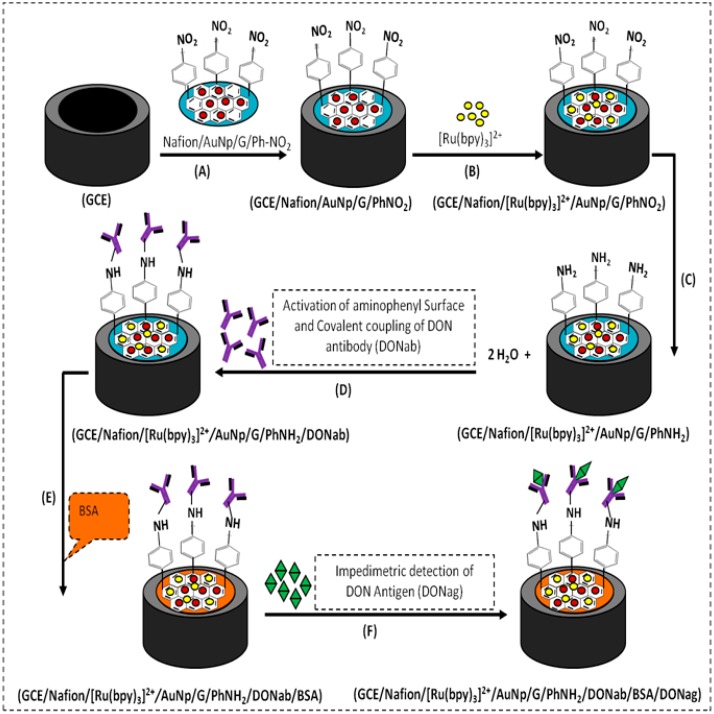
Schematic representation for stepwise fabrication of the label-free impedimetric deoxynivalenol immunosensor.

The sensor platform was prepared in stages as illustrated in Figure 3. (A) Nafion stock solution was diluted with 1:1 water to methanol mixture to yield 1% (v/v) solution and then AuNp/G/PhNO_2_ was dispersed in the 1% (v/v) Nafion solution by ultra-sonication for 30 min to form 1 mg·mL^−1^ uniform nano-composite. 4 μL of it was drop-casted on the surface of a pre-cleaned GCE and excess solvent was allowed to evaporate to dryness in the open at room temperature to form a thin film; (B) The Nafion/AuNp/G/PhNO_2_ modified GC electrode was placed in 1 mM Ru(bpy)_3_Cl_2_ aqueous solution for 2 h to incorporate [Ru(bpy)_3_]^2+^ via electrostatic and ion-exchange interactions, forming a thin film modified glassy carbon electrode depicted as GCE/Nafion/[Ru(bpy)_3_]^2+^/AuNp/G/PhNO_2_.

### 2.8. Immobilization of Deoxynivalenol Antibody

Before immobilization of antibody, (C) the modified electrode was first reduced electrochemically to generate an aminophenyl surface in 0.1 KCl solutions by two cyclic voltammetric scans over potential range 0 to −1200 mV at 50 mV/s [18–20]; (D) the electrode was then rinsed out from the sides after reduction using deionised water and the amine on the electrode surface was activated by incubation for 60 min with 1:1 mixture of 0.5 mM EDC and 8 mM NHS.

After rinsing the electrode from the side with 0.1 M PBS (pH 7.2) solution, 30 μL of 0.2 μg/μL (in 0.1 M PBS, pH 7.2) monoclonal deoxynivalenol antibody (DONab) was spread on the electrode surface and incubated for 30 min at room temperature; (E) the electrode was again rinsed from the side using 0.1 M PBS (pH 7.2) solution to remove any physically bound antibody and then 10 μL of BSA was spread on the electrode surface to block nonspecific binding sites for 30 min at room temperature and rinsed with PBS (pH 7.2) before making impedimetric measurements; (F) the immunosensor which is depicted as GCE/Nafion/[Ru(bpy)_3_]^2+^/AuNp/G/PhNH_2_ /DONab/BSA was incubated for 30 min in aliquots of a pH 7.2 MPBS solution containing various concentrations of DONag and measured impedimetrically [20–22]. All experiments were carried out at room temperature (see Figure 3).

## 3. Results and Discussion

### 3.1. Electro-Reduction of Nitrophenyl Group to Aminophenyl Group

**Figure 4.**
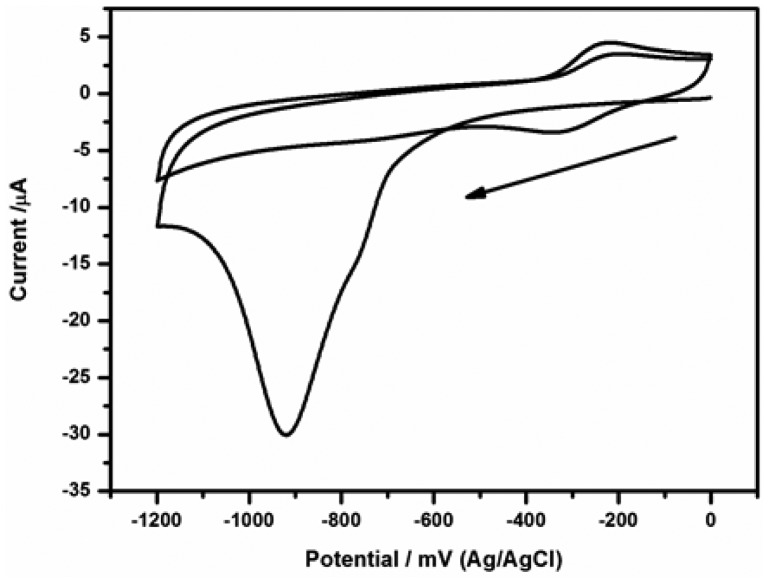
Electrochemical reduction of 4-nitrophenyl group to 4-aminophenyl on the electrode surface.

The modified electrode was first reduced electrochemically to obtain a modified film of 4-aminophenyl on the electrode surface in 0.1 KCl solutions by two cyclic voltammetric scans over potential range 0 to −1200 mV at scan rate of 50 mV/s. The modified electrode showed irreversible voltammogram with a reduction peak attributable to 4-nitrophenyl (-PhNO_2_) to 4-aminophenyl (-PhNH_2_) reduction at −920 mV (Ag/AgCl), see Figure 4. During the second scan, the peak observed at −920 mV in the first scan disappeared, indicating that nearly all the electroactive -PhNO_2_ were reduced in the first scan. However, the reduction of -PhNO_2_ to -PhNH_2_ is incomplete as evidenced by the reversible couple that appeared at *E*_½_ = −250 mV (Ag/AgCl). This couple is assigned to the aminophenyl/nitrosophenyl inter-conversion [23,24].

### 3.2. CV Behaviour of Electrochemically Reduced Modified Electrodes

**Figure 5.**
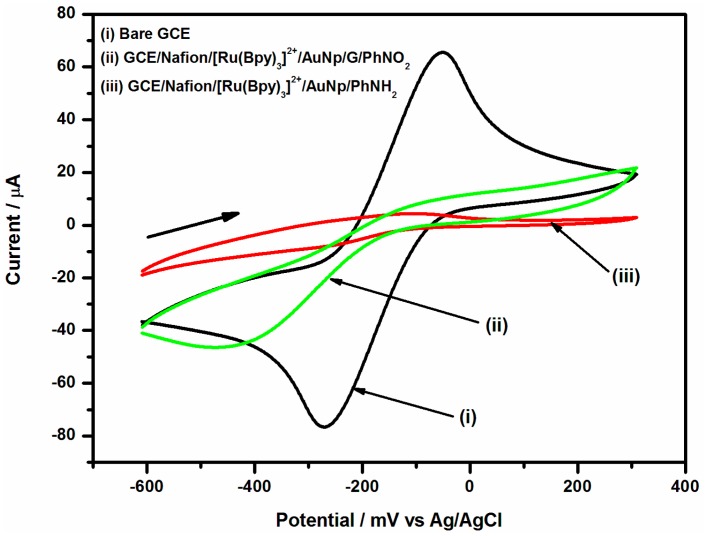
CV of (i) bare GCE, (ii) GCE/Nafion/[Ru(bpy)_3_]^2+^/G/PhNO_2_ and (iii) GCE/Nafion/[Ru(bpy)_3_]^2+^/AuNp/G/PhNH_2_ recorded in 5 mM [Ru(NH_3_)_6_]^2+/3+^.

Once the p-aminophenyl surface layer was formed, the electrochemical behaviour of the modified GC/Nafion/[Ru(bpy)_3_]^2+^/AuNp/G/PhNH_2_ electrode surface was investigated by cyclic voltammetry in the presence of [Ru(NH_3_)_6_]^2+/3+^ redox couple. Figure 5 shows the cyclic voltammogram for bare GC electrode, GC/Nafion/[Ru(bpy)_3_]^2+^/AuNp/G/PhNO_2_ electrode and for GCE/Nafion/[Ru(bpy)_3_]^2+^/AuNp/G/PhNH_2_ electrode, in the presence of the [Ru(NH_3_)_6_]^2+/3+^ redox couple. The data clearly show that the characteristic oxidation/reduction waves of the [Ru(NH_3_)_6_]^2+/3+^ redox couple was completely suppressed at the GCE/Nafion/[Ru(bpy)_3_]^2+^/AuNp/G/PhNH_2_ electrode in curve (iii). This is expected for completely blocked 4-aminophenyl surface [19,21]. The suppression of the redox peaks at CV curve (iii) is attributed to repulsion forces between the positively charged redox probe and the positively charged (after reduction) amine (-PhNH_2_^+^) groups.

### 3.3. CV Behaviour of Stepwise Modification Processes of the Immunosensor

**Figure 6.**
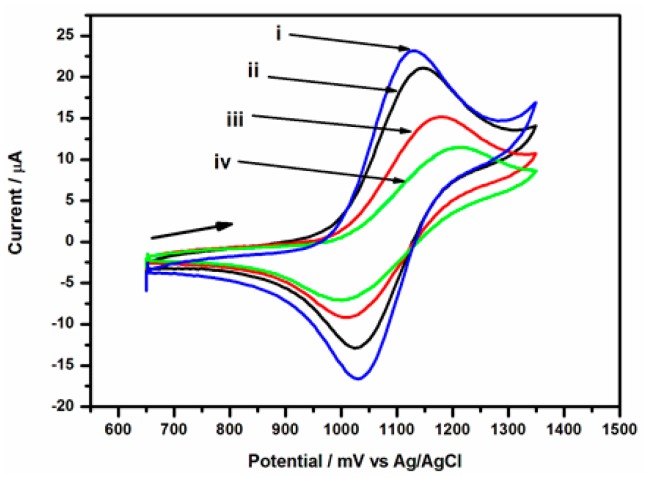
CV response of different immobilisation steps: (i) GCE/Nafion/[Ru(bpy)_3_]^2+^/AuNp/G/PhNH_2_, (ii) after immobilization of DON antibody, (iii) after blocking nonspecific binding sites with BSA and (iv) after immobilization and running in standard DON solutions at scan rate of 50 mV/s, over potential range of 650–1350 mV.

The electrochemical behaviour of various stages in the modification of GCE/Nafion/[Ru(bpy)_3_]^2+^/AuNp/G/PhNH_2_/DONab immunosensor was studied by cyclic voltammetry (See Figure 6). It was observed that the immobilization of deoxynivalenol antibody (DONab) on GCE/Nafion/[Ru(bpy)_3_]^2+^/AuNp/G/PhNH_2_ (see curve i) lead to a decrease in peak current of the [Ru(bpy)_3_]^2+^ redox probe in GCE/Nafion/[Ru(bpy)_3_]^2+^/AuNp/G/PhNH_2_/DONab (see curve ii). This is due to decreased electron transfer capability of the DONab modified electrode.

This result confirms that the immobilized DONab formed an insulating layer on the electrode and perturbs the interfacial electron transfer considerably. After BSA was immobilized to block the remaining active cites in order to avoid any nonspecific adsorption, a further decrease of the peak currents was observed with the fact that BSA insulates the conductive support and hinders the transmission of electrons toward the electrode surface further (curve iii). These results indicated that the immunosensor was successfully modified as premeditation. The peak current reduced further after the immunosensor was applied in detecting DON from the standard solution (curve iv).

### 3.4. Chronoamperometric Behaviour of Stepwise Modification Processes of the Immunosensor

The electrochemical behaviour of the stepwise modification processes of GCE/Nafion/[Ru(bpy)_3_]^2+^/AuNp/G/PhNH_2_/DONab immunosensor was further investigated by chronoamperometry. The experiments were done in a two-step potential mode, with 2 s quiet time, and pulse width of 500 ms in a quiescent condition. The choice of potential steps was made from the cyclic voltammogram at 50 mV/s of the sensor platform in 0.1 M PBS (pH 7.2).

**Figure 7.**
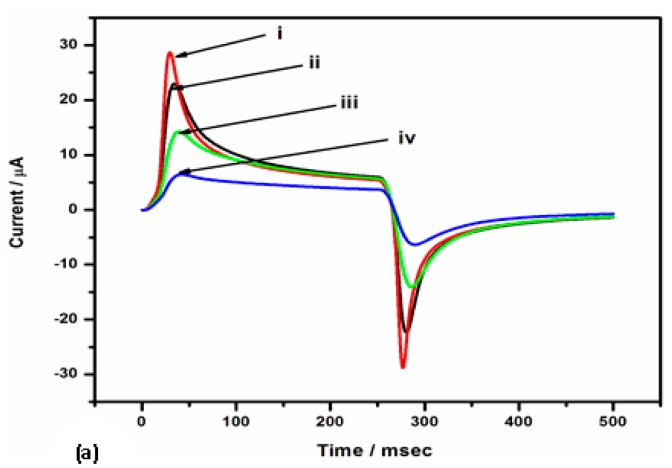

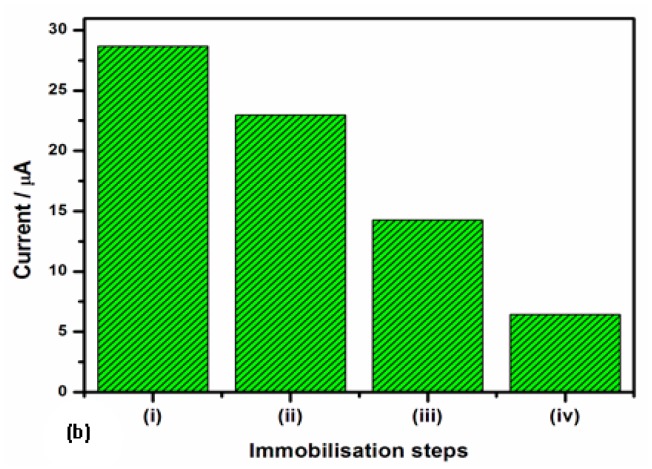
(**a**) Chronoamperometric responses, and (**b**) bar chart for the chronoamperograms of different immobilisation steps: (i) GCE/Nafion/[Ru(bpy)_3_]^2+^/AuNp/G/PhNH_2_, (ii) after immobilization of DON antibody, (iii) after blocking nonspecific binding sites with BSA and (iv) after immobilization and running in standard DON solutions.

The Faradaic chronoamperometric measurements were in good agreement with the CV measurements. It could be observed from the chronoamperogram and bar chart in Figure 7 respectively that the current reduced with each modification step. Following the observed blocking effect of the -PhNH_2_ film in the previous sections, this indicates that a further blocking effect was observed after the covalent immobilization of the antibody and blocking of nonspecific binding sites with BSA.

### 3.5. Optimisation of Assay Condition

#### 3.5.1. Effect of Antibody Concentration

**Figure 8.**
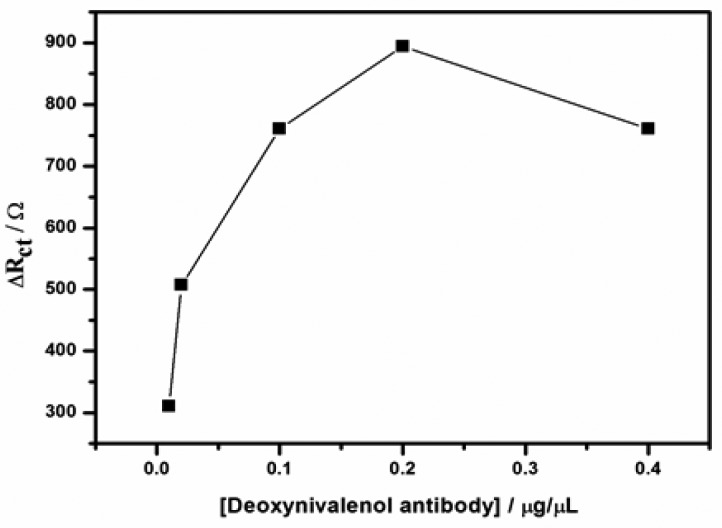
The plot of R_ct_ values *versus* deoxynivalenol antibody concentrations.

The electrochemical responses of the immunosensor to various concentrations of DONab applied in the immobilisation step were determined by impedance spectroscopy. Different dilutions of DONab were prepared (1:10; 1:50; 1:100, 1:200 and 1:400 v/v, corresponding to 0.01, 0.02, 0.1, 0.2 and 0.4 μg/μL). 30 μL of each concentration was drop coated onto the modified electrode to obtain GCE/Nafion/[Ru(bpy)_3_]^2+^/AuNp/G/PhNH_2_/DONab, and then impedimetric measurements were performed in presence of 30 ng/mL DONag MPBS solution (pH 7.2). The highest R_ct_ was obtained with 0.2 μg/μL (Figure 8), indicating the optimal formation of immune-complexes and inhibition. Thus, concentration of 0.2 μg/μL was selected for all experiments.

#### 3.5.2. Effect of pH

**Figure 9.**
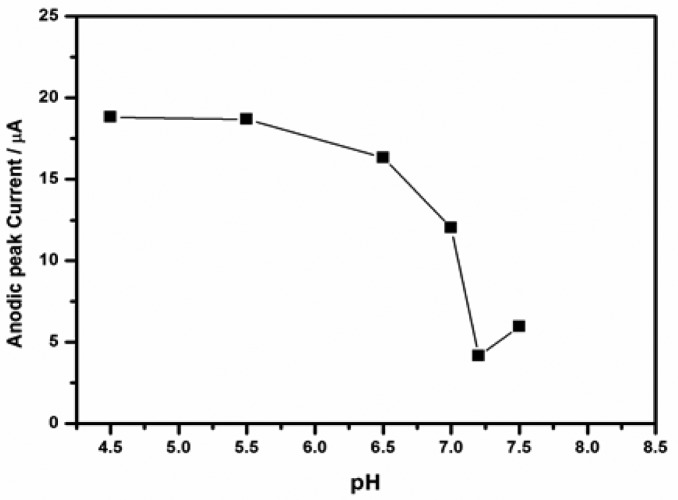
The plot of anodic peak currents from CV *versus* solution pH.

In order to evaluate the influence of the pH on the immunosensor performance, it was tested by cyclic voltammetric technique in a series of PBS working solutions with pH ranging from 4.5 to 7.5. Figure 9 shows the pH effect of the detection solution on the immunosensor. The current responses in presence of 30 ng/mL DONag were monitored. There was no significant change in the peak currents between pH 4.0–6.5 indicating that the bioactivity of antigen and antibody was poor or absent in the acidic solution. Conversely, a drastic drop in peak current was observed at pH 7.0 and the lowest current was at pH 7.2. This could be attributed to the formation of immunocomplexes by the binding of DONab and DONag which led to inhibition of electron flow. Therefore PBS of pH 7.2 was adopted as the incubation solution considering the response of the immunosensor.

#### 3.5.3. Effect of Incubation Time

**Figure 10.**
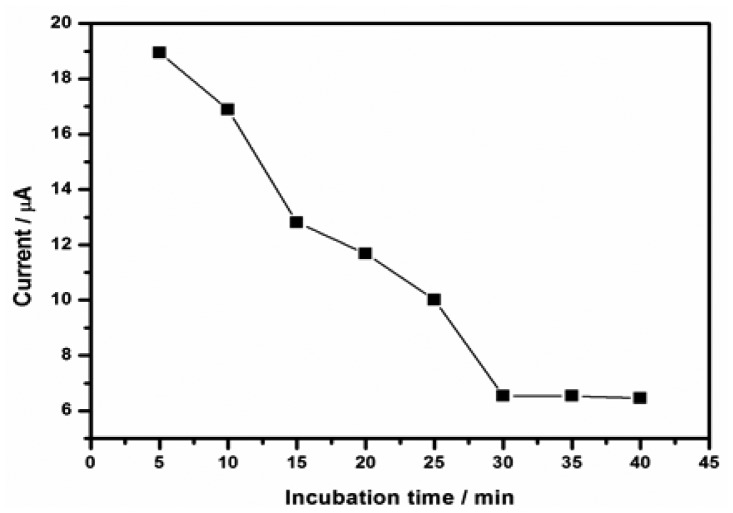
The plot of peak currents from chronoamperograms versus incubation time.

Incubation time for the antigen–antibody interaction greatly influence the sensitivity of the developed immunosensor. The chronoamperometric technique was used to investigate the current responses of various immunochemical incubation times (from 5 to 40 min) of the immunosensor. The immunosensor was incubated in 30 ng/mL DONag MPBS solution (pH 7.2). It was observed that the current decreased as the incubation time increased and levelled off after 30 min, indicating the optimal formation of immune-complexes, therefore an incubation time of 30 min was adopted for subsequent analysis (see Figure 10).

### 3.6. Performance of GCE/Nafion/[Ru(bpy)_3_]^2+^/AuNp/G/PhNH_2_/DONab/BSA Immunosensor

#### 3.6.1. Calibration Curve

**Figure 11.**
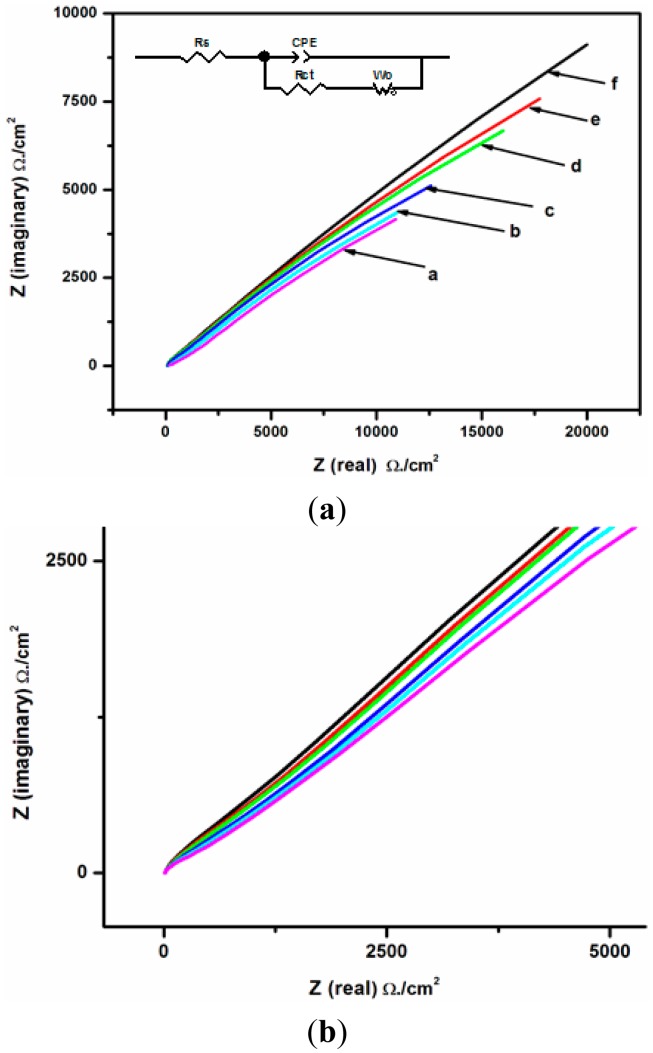
(**a**). EIS responses of GCE/Nafion/[Ru(bpy)_3_]^2+^/AuNp/G/PhNH_2_/DONab/BSA immunosensor to standard DONag solutions: a, b, c, d, e and f represents 0, 6, 12, 18, 24 and 30 ng/mL DON antigen respectively; (**b**). Expanded EIS spectra responses of GCE/Nafion/[Ru(bpy)_3_]^2+^/AuNp/G/PhNH_2_/DONab/BSA immunosensor to standard DONag solutions indicating the characteristic R_ct_ semi-circle over the high frequency range and a straight line over the low frequency range.

The immunosensor was incubated for 30 min in aliquots of a pH 7.2 methanolic phosphate buffer saline (MPBS) solution containing various concentrations of DONag. These different concentrations of DONag were tested and similar impedance spectra with increasing R_ct_ as the concentration increased were obtained. Figure 11 shows the Nyquist diagrams of the immunosensor obtained after incubation in increasing concentrations (0, 6, 12, 18, 24 and 30 ng/mL) of DONag. The observed increase in R_ct_ values with increasing DONag concentrations implies that more DONag are being bound to the interface and also indicates that the binding of DONag to the DONab immunosensor inhibits electron transfer at the sensing interface [21,25]. This reflected as an increase in charge transfer resistance in both imaginary and real impedance as shown in Figure 11.

**Figure 12.**
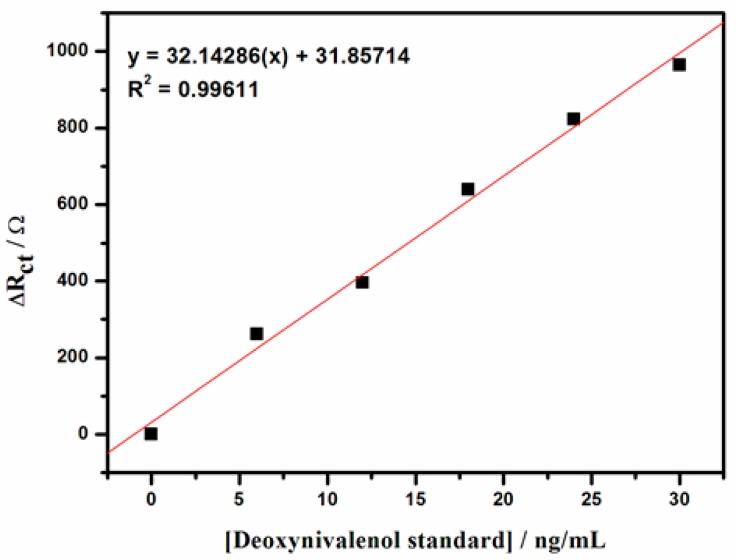
The calibration plot of EIS detection data of the immunosensor.

The equivalent electrical circuit presented in the Figure 11a insert was used to fit the responses of the immunosensor to various standard DONag solutions in order to obtain the value of the charge transfer resistance at each concentration. Figure 11b shows **e**xpanded EIS spectra responses of GCE/Nafion/[Ru(bpy)_3_]^2+^/AuNp/G/PhNH_2_/DONab/BSA immunosensor to standard DONag solutions: a, b, c, d, e and f represents 0, 6, 12, 18, 24 and 30 ng/mL DON antigen respectively. In order to compare the different electrode measurements under the equivalent circuit conditions and obtain the normalised values that were plotted in Figure 12, the R_ct_ value for the blank (0 ng/mL DONag) was subtracted from the R_ct_ values of DONag concentrations. A linear relationship between the electron transfer resistance and DONag concentrations ranged from 0 to 30 ng/mL. When DONag was increased beyond 30 ng/mL, the change in impedance spectroscopy gradually levelled out indicating that all the available binding sites on the sensor were occupied by the DONag. The detection limit of 0.2994 μg/L was obtained on the basis of 3× S.D. of determination of the zero standards [26] and the sensitivity is 32.14286 ΩL/ng.

#### 3.6.2. Stability, Reproducibility, Repeatability, and Selectivity of the Immunosensor

The stability of the GCE/Nafion/[Ru(bpy)_3_]^2+^/AuNp/G/PhNH_2_/DONab/BSA immunosensor was assessed by impedimetrically measuring 30 ng/mL of DONag in MPBS solution at daily intervals for 5 days with the same sensor. Minimal detectable loss of the activity was observed in comparing the daily signals with the signal of freshly prepared immunosensor. The result shows that the immunosensor is 80.3% stable. The sensor was not measured after this period but longer term stability may be expected due to the strong covalent attachment of the antibody to the modified electrode which could prevent the antibody from leaking out from the surface.

The reproducibility of the immunosensor was investigated by measuring 30 ng/mL of DONag in MPBS solution using five similarly prepared immunosensors. Relative standard deviation of 6.5% was calculated. Such good precision reflects the reproducibility of the electrode modifications, immune-binding and impedimetric procedures. Its repeatability was investigated by five successive measurements of 30 ng/mL of DONag in MPBS solution using the same immunosensor. The relative standard deviation (RSD) for the five parallel impedimetric measurements is 6.9%. This result indicates that the repeatability of the sensor was within experimental error.

**Figure 13.**
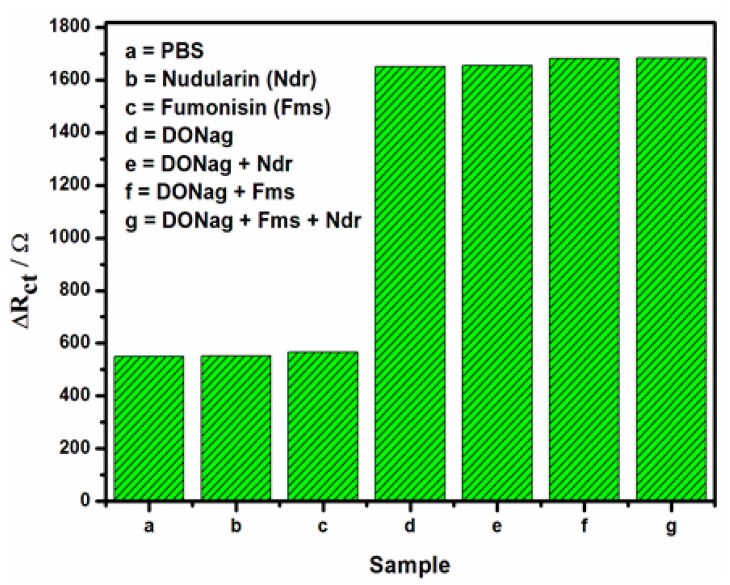
The selectivity chart of GCE/Nafion/[Ru(bpy)_3_]^2+^/AuNp/G/PhNH_2_/DONab immunosensor.

To investigate the selectivity of the immunosensor, it was incubated in 30 ng/mL DONab standard solution containing different interfering agents such as nudularin and fumonisin. No remarkable change of R_ct_ was observed in comparison with that which contains only DONab except for the slight increase in R_ct_ observed when fumonisn was added. The immunosensor was also incubated in 30 ng/mL fumonisin and 30 ng/mL nudularin standard solutions prepared in MPBS (pH7.2) solution. The same R_ct_ value was obtained for 30 ng/mL nudularin standard solution compared to that of the blank MPBS (pH 7.2) solution while a slight increase in R_ct_ value was observed for 30 ng/mL fumonisin (Figure 13). These results show that the immunosensor has good selectivity.

### 3.7. Detection of DONag Standards by Veratox ELISA Test Kit

**Figure 14.**
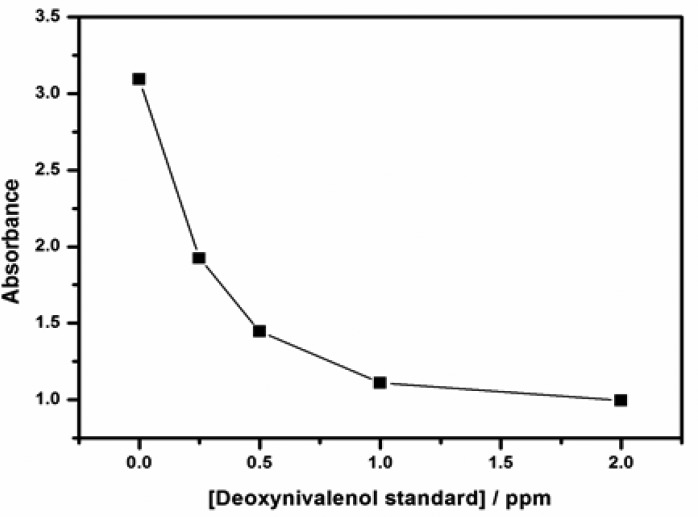
The detection plot of DONag standards by ELISA.

Various standard concentrations of DONag were tested using ELISA method to valid the fabricated immunosensor. Veratox for DON 5/5 is a competitive direct enzyme-linked immunosorbent assay (CD-ELISA) which is intended for quantitative analysis of deoxynivalenol in grains and grain products, and it allows the user to detect DONag concentrations in parts per million (ppm). The concentration range of DONg standard for the Veratox ELISA was between 0.25–1.99 ppm. A standard calibration curve of absorbance *versus* DONag concentrations was plotted as shown in Figure 14. The sensitivity obtained from slope of the linear part of the graph was calculated to be 0.041 mg/L and the limit of detection was 2.1 ppm. These values represent a better feedback in detecting DONag and make the use of ELISA an appropriate technique in validating the fabricated immunosensor.

### 3.8. Real Sample Studies

The feasibility of the proposed impedimetric immunosensor for real sample analysis was investigated by detecting DONag in extracted certified reference materials of wheat; roasted coffee and corn following the extraction procedure described by Veratox Elisa Kit manual. The extracts from the reference materials were comparably analysed with GCE/Nafion/[Ru(bpy)_3_]^2+^/AuG/PhNH_2_/DONab and Veratox 5/5 ELISA kit and the results are shown in Table 1. The detected DONag values from the different extracts using the immunosensor were comparable to those obtained with Veratox ELISA as well as the quantity advertised by the vendor.

## 4. Conclusions

A gold nanoparticles-dotted 4-nitrophenylazo-functionalized graphene (AuNp/G/PhNO_2_) nanocatalyst was successfully used to fabricate a label-free electrochemical immunosensor for detection and determination of the mycotoxin deoxynivalenol in cereals. The impedimetric detection technique was employed and under the optimized conditions, the formation of immunocomplexes inhibited electron flow and increased the charge transfer resistance of the sensing interface linearly. The detection range of the immunosensor for deoxynivalenol (DONag) in standard samples was 6–30 ng/mL. Its sensitivity and detection limit were 32.14 ΩL/ng and 0.3 μg/L respectively, which were better than those reported in the literature and compare favorably with the ELISA result. The immunosensor is very simple to operate by direct immunoreaction detection without any label and competitive assay. The sensitivity of this sensor ensures its use, not only in the laboratory but also in the field. The immunosensor can be fabricated easily and it exhibits promising application prospects as a novel electrochemical sensor.

## Figures and Tables

**Table 1.** DON content of corn, wheat and roasted coffee certified reference materials

**Certified Reference Materials**	**DON Immunosensor (ppm)**	**ELISA (ppm)**	**Vendor (ppm)**
Wheat	0.3	0.2	0.71
Corn	0.92	1.1	1.88
Roasted coffee	0.19	0.24	0.77
